# Mitochondrial Dysfunction and Stress Responses in Alzheimer’s Disease

**DOI:** 10.3390/biology8020039

**Published:** 2019-05-11

**Authors:** Ian Weidling, Russell H. Swerdlow

**Affiliations:** 1 University of Kansas Alzheimer’s Disease Center, Fairway, KS 66205, USA; iweidling2@kumc.edu; 2 Department of Integrated and Molecular Physiology, University of Kansas Medical Center, Kansas City, KS 66160, USA; 3 Department of Neurology, University of Kansas Medical Center, Kansas City, KS 66160, USA; 4 Department of Biochemistry and Molecular Biology, University of Kansas Medical Center, Kansas City, KS 66160, USA

**Keywords:** Alzheimer’s disease, eIF2α, metabolism, mitochondria, proteostasis, stress response

## Abstract

Alzheimer’s disease (AD) patients display widespread mitochondrial defects. Brain hypometabolism occurs alongside mitochondrial defects, and correlates well with cognitive decline. Numerous theories attempt to explain AD mitochondrial dysfunction. Groups propose AD mitochondrial defects stem from: (1) mitochondrial-nuclear DNA interactions/variations; (2) amyloid and neurofibrillary tangle interactions with mitochondria, and (3) mitochondrial quality control defects and oxidative damage. Cells respond to mitochondrial dysfunction through numerous retrograde responses including the Integrated Stress Response (ISR) involving eukaryotic initiation factor 2α (eIF2α), activating transcription factor 4 (ATF4) and C/EBP homologous protein (CHOP). AD brains activate the ISR and we hypothesize mitochondrial defects may contribute to ISR activation. Here we review current recognized contributions of the mitochondria to AD, with an emphasis on their potential contribution to brain stress responses.

## 1. Introduction

Sporadic Alzheimer’s disease (AD) brains possess profound mitochondrial defects, including changes in number, morphology, and enzyme activity [1,2,3]. Mitochondrial dysfunction in AD is not restricted to the nervous system. Systemic mitochondrial defects occur in AD patients compared to controls [4,5]. Metabolic defects occur alongside mitochondrial abnormalities in AD, providing early markers of disease progression [6]. Mitochondrial dysfunction may contribute to hallmark AD pathology and stimulate stress response pathways.

## 2. AD Brain Hypometabolism

Brain glucose uptake studies provided some of the earliest evidence for AD metabolic defects. Changes in cerebral glucose utilization occur during AD, demonstrated by numerous studies using [18F]-2-fluoro-2-deoxy-D-glucose (FDG) coupled with positron emission tomography (PET) [7,8,9,10]. In these studies, researchers administer radiolabeled FDG to patients intravenously. Cells take FDG up through glucose importers and subsequently phosphorylate FDG via hexokinase. Unlike glucose, FDG cannot be processed further by glycolytic enzymes and accumulates within the cell. Cells taking up more radiolabeled FDG display a stronger PET signal [11]. AD patients consistently display reduced cerebral PET signals following [18F] FDG administration suggesting reductions in glucose uptake and neuronal activity [12,13,14].

Classically, AD brains display decreased temporo-parietal glucose uptake in both hemispheres [15]. Studies have attempted to correlate numerous AD pathological changes with cognitive decline. FDG PET studies show cerebral glucose utilization correlates reasonably well with cognitive decline. Amyloid plaques, on the other hand, correlate poorly with cognitive decline, while neurofibrillary tangles (NFTs) show better correlation [16]. Although a definitive diagnosis of Alzheimer’s disease requires the presence of amyloid plaques and NFTs, brain hypometabolism may provide a sensitive and early marker of neurodegeneration [17]. 

Decreased cerebral glucose utilization occurs early in AD and could prove useful diagnostically [6,18,19]. Studies performing FDG PET analysis on patients with “very early Alzheimer’s disease” found changes in glucose uptake. The study divided participants into a very early Alzheimer’s disease group and an age-matched control group based on mini-mental state exam performance. The very early Alzheimer’s disease group displayed brain region specific decreases in glucose utilization relative to age-matched controls. Early glucose uptake deficits presented most prominently in the posterior cingulate cortex (PCC) and cinguloparietal transition regions. Reports describe neurodegeneration in these regions in neuropathologically confirmed AD cases. Reduced glucose utilization in the very early Alzheimer’s disease brain did not correlate with AD pathology. Neuropathological examination of very early Alzheimer’s disease brains found NFT accumulation in medial and inferior temporal cortex but not in the PCC. It is interesting to note that metabolic deficiencies occur in the absence of AD pathology, suggesting NFTs and plaques do not need to be present for reduced glucose utilization to occur [13]. Longitudinal FDG PET studies, followed up with neuropathological diagnosis, demonstrate further AD specific changes in brain metabolism. This study improved upon prior work by confirming eventual AD diagnosis. The results support brain glucose utilization as a potential tool in AD diagnosis [20]. Several studies show metabolic defects can be detected long before the onset of cognitive decline [21,22]. Meta-analysis of studies evaluating FDG-PET for AD diagnosis shows FDG-PET performs better in diagnosing AD than current diagnostic methodologies [23]. Brain hypometabolism’s early appearance and correlation with dementia in AD patients suggests altered metabolism is intimately linked with disease progression.

A clear association between brain hypometabolism and dementia exists but researchers do not understand why AD brains display reduced glucose utilization. Glucose transporter studies in AD brains provide one potential explanation for decreased glucose utilization. Glucose transporters move glucose across cell membranes and into the cytoplasm. Neurons import glucose mainly through GLUT3, while astrocytes import glucose mainly through GLUT1 [24]. Both GLUT1 and GLUT3 protein levels decrease in AD brain and these changes in GLUT1 and GLUT3 persist after correcting for cell death. For this reason, Simpson et al. argue glucose transporter loss contributes to neurodegeneration [25]. Further studies found reduced glucose transporter levels at the blood-brain barrier in AD brains, another likely contributor to decreased glucose uptake [26]. Decreasing glucose transporter levels speak to broad metabolic defects in AD brain. Mitochondrial dysfunction likely contributes to these broad and general AD metabolic defects.

## 3. AD Mitochondrial Defects

Altered metabolism in AD coincides with numerous mitochondrial changes. AD platelet cytochrome oxidase (COX) activity studies provided early evidence for mitochondrial dysfunction. Parker et al. showed altered COX activity in AD platelets, later extending their findings to AD brain tissue [4,27]. At this time, they postulated mitochondrial DNA (mtDNA) alterations may trigger AD COX deficiencies [28]. An additional study characterized AD mitochondrial complex I and II–III activities, finding no consistent activity changes in various brain areas. However, the study confirms decreased COX activity in multiple AD cortical brain regions [29]. Mitochondrial tricarboxylic acid (TCA) cycle enzymes also display altered activity in AD brain. Post-mortem AD brain activity assays reveal increases and decreases in TCA enzyme activities. Among mitochondrial enzyme activities, clinical decline correlates most closely with changes in pyruvate dehydrogenase complex activity [30]. Defects in numerous mitochondrial enzymes exist in AD brain, likely contributing to metabolic abnormalities.

Further changes in mitochondrial enzymes exist in AD. Post-mortem AD brain tissue analysis finds that COX subunits decrease during disease progression. In one study, the authors analyzed COXIV (nuclear-encoded) and COXII (mitochondrial-encoded) subunit levels in cerebellar Purkinje neurons, an area relatively preserved in AD subjects compared to age-matched controls. The study found decreased COXIV and COXII protein levels in AD Purkinje neurons relative to age matched controls, as well as COX subunit reductions in aged controls relative to young controls. Based upon this finding, the authors argue COX deficiency occurs during normal aging and accelerated COX deficiency contributes to AD progression [31]. Cottrell et al. [32] discovered increased COX deficient neurons in the AD hippocampus. The study examined COX and mitochondrial complex II (succinate dehydrogenase) levels in individual cells via immunohistochemistry (IHC). Neurons containing drastically reduced COX levels with normal succinate dehydrogenase levels were classified as COX deficient [32]. The specific reduction in COX levels relative to succinate dehydrogenase suggests mitochondrial mass is maintained while COX is preferentially depleted. Subsequent studies correlated AD pathology with COX deficiency. Correlational studies revealed COX deficient neurons contain decreased NFTs relative to surrounding COX positive neurons. The study found no correlation between COX levels and plaque burden [33]. 

While the AD hippocampus contains many COX deficient neurons, studies also observe AD neurons displaying increases in COX and mtDNA. In AD neurons with increased COX and mtDNA, lysosomal structures tend to accumulate mitochondrial components. These findings suggest increases in COX and mtDNA do not reflect increased intact mitochondria. Instead, mtDNA and COX accumulation likely signals deficient mitochondrial degradation [34]. AD neurons upregulate lysosomal components early in the disease process. AD neurons increase lysosomal protease (cathepsin D) mRNA and protein with concomitant lysosomal accumulation [35]. Furthermore, diseased neurons accumulate autophagosomes at a high level, suggesting either an increased autophagic rate, decreased autophagosome maturation, or both [36]. Disruptions to autophagy and lysosomal degradation likely contribute to AD mitochondrial defects. Defective mitochondria generally undergo selective degradation through an autophagosome dependent process known as mitophagy. To begin the process of mitophagy, autophagic vacuoles surround and envelope mitochondria. Autophagic vacuoles containing mitochondrial components then acidify, maturing to lysosomes, and degrading their contents [37]. Mitophagy maintains a healthy mitochondrial pool, so disruptions in this process compounds other mitochondrial defects [38]. Mitophagy is altered in AD, as studies observe increased mitochondria-lysosome associations. 

AD mitochondria also display alterations in morphology. AD brain electron microscopy (EM) studies reveal changes in mitochondrial physical structure. Numerous AD brain regions display increased variability in mitochondrial shape and disrupted cristae, as well as decreased mitochondrial surface area [39]. Mitochondrial morphology relies on fission and fusion processes. Mitochondrial fission and fusion defects occur in AD and likely contribute to morphological changes. Zhang et al. [40] performed three-dimensional (3D) reconstruction of serial AD hippocampal EM sections. 3D EM revealed a novel AD mitochondrial morphology termed “mitochondria on a string” (MOAS). Earlier methodologies could not detect this morphological feature, likely classifying MOAS as fragmented mitochondria. MOAS likely form when fission machinery malfunctions. The authors propose AD bioenergetic defects inhibit fission machinery, triggering mitochondrial morphology changes [40]. Additional studies suggest AD disrupts fusion and fission. Wang et al. describe altered mitochondrial localization in AD pyramidal neurons along with altered fusion and fission proteins [41]. Experiments also show that amyloid beta can cause fusion and fission defects, and inhibiting mitochondrial fission proves beneficial in AD mouse models. Mutant amyloid precursor protein overexpression in primary mouse hippocampal neurons altered fusion and fission genes and disrupted mitochondrial structure [42]. Additionally, amyloid beta treatment in neuronal cells caused dynamin related protein 1 (Drp1) phosphorylation and increased mitochondrial fission. A mitochondrial fission inhibitor reduced reactive oxygen species (ROS) and reduced mitochondrial dysfunction caused by amyloid beta treatment [43,44]. Studies of mitochondrial fission in AD models also suggest that mitochondrial fission favors cell death. In fact, amyloid beta oligomers trigger mitochondrial fragmentation and subsequent cell death via the loss of a mitochondrial fusion factor [45]. Inhibiting mitochondrial fission in an AD mouse model decreased brain pathology and improved memory, as well as synaptic connections, suggesting mitochondrial fission inhibitors may have therapeutic potential [46]. Mitochondrial morphological changes in AD speak to widespread mitochondrial dysfunction.

Whether metabolic and mitochondrial defects represent a cause or consequence of AD remains controversial. Numerous groups propose mitochondrial dysfunction initiates AD pathological cascades and therapeutics should target mitochondrial dysfunction [1,47,48,49]. The cause for mitochondrial dysfunction in AD remains unclear, however. Many theories explaining AD mitochondrial dysfunction exist. Groups viewing mitochondrial dysfunction as a primary event in AD progression point to mtDNA as a potential disease driver [28,50]. Groups viewing mitochondrial dysfunction as a disease consequence propose AD pathology, namely amyloid protein and tau tangles, initiates mitochondrial dysfunction [51,52,53]. Still other groups propose defective mitochondrial quality control and oxidative damage contributes to mitochondrial dysfunction [34]. Each of these mechanisms may in fact contribute to AD mitochondrial dysfunction and, increasingly, mitochondrial function is viewed favorably as a therapeutic target. 

## 4. Role of Mitochondrial DNA in AD

Somatic mtDNA mutations may contribute to AD and mtDNA inheritance may influence AD risk. Mitochondrial function relies on coordinated expression of genes from the nuclear and mitochondrial genomes. Inherited mtDNA polymorphisms cause a range of disorders known as primary mitochondrial diseases. Many of these diseases primarily affect cognition, demonstrating that neurons possess high sensitivity to mitochondrial defects. One of the most widely recognized mtDNA deletions, a 4997 bp deletion called the “common deletion”, increases in the brain during normal aging. Common deletion rates increase most drastically in regions with high metabolic activity, causing some to speculate that mtDNA somatic deletions dispose individuals to neurological disease [54]. AD mtDNA alterations surpass those observed in age-matched controls. High common deletion rates occur early in the AD cortex. As AD patients reach age 80, however, common deletion rates typically decline. The opposite trend exists in age-matched control cortex, with low common deletion rates early and increasing rates as individuals age [55,56].

AD brains also display increased mtDNA oxidative damage. Interestingly, mtDNA oxidative damage occurs most heavily in the parietal lobe, which displays early and consistent hypometabolism [57]. Increased oxidative damage correlates with mitochondrial dysfunction. For this reason, researchers speculated that AD oxidative damage favors mtDNA mutations. Indeed, mtDNA control region mutations increase in AD frontal cortex. Increased control region mutations associate with decreased mtDNA transcription and replication [58]. Subsequent analyses utilizing next generation sequencing (NGS) discovered increased AD hippocampal mtDNA point mutations. However, the authors conclude AD point mutations likely stem from mtDNA replication errors rather than oxidative damage [59].

AD inheritance pattern studies implicate mtDNA inheritance as an AD risk factor. In a group of families with one AD affected parent and two affected siblings, Edland et al. discovered increased AD rates among individuals with a maternal AD history [60]. These findings suggest AD favors a maternal inheritance pattern. Maternal AD history also increases risk for brain hypometabolism, potentially increasing AD risk [61]. Groups propose mtDNA inheritance explains AD’s subtle but identifiable maternal inheritance predominance [48]. mtDNA largely passes from mother to child, and therefore AD’s bias towards maternal inheritance is consistent with mtDNA influencing AD risk. 

Cytoplasmic hybrid (cybrid) studies provide further evidence mtDNA contributes to AD mitochondrial abnormalities. Cybrid generation occurs by repopulating cells lacking mtDNA (ρ0) with exogenous mtDNA. Exogenous mtDNA often comes from patient platelets, allowing creation of cybrids containing AD patient mtDNA. Cybrids, therefore, effectively model AD mitochondrial function on a stable nuclear background. AD cybrids recapitulate numerous AD features. Initial AD cybrid studies demonstrate COX activity deficits that recapitulate those of AD patient mitochondria. AD cybrid COX deficits provide strong evidence that mtDNA contributes to AD mitochondrial defects [62,63]. Further studies suggest mtDNA deletions contribute to AD hippocampal COX deficiency. As referenced earlier, COX deficient neurons increase in the AD hippocampus. COX deficient AD neurons contain increased mtDNA deletions, suggesting mtDNA deletions contribute to COX deficiency [64]. Additional AD cybrid studies describe enlarged, swollen mitochondria with reductions in membrane potential and increases in ROS and antioxidant enzymes [63,65]. AD cybrid studies also suggest mitochondrial dysfunction can drive changes in AD neuropathology.

AD cybrids display amyloid changes reminiscent of those observed in AD and possess increased sensitivity to amyloid beta fragments. AD cybrids release amyloid beta at greater rates than controls. Furthermore, AD cybrids contain increased intracellular amyloid beta. Elevations in amyloid beta coincide with increased cytochrome c release and caspase-3 activity, suggesting cell death pathway activation may contribute to elevated amyloid beta [66]. AD cybrids treated with amyloid beta display enhanced cell death pathway activity compared to control. Mitochondrial membrane potential, cytochrome c release and caspase 3 activity all change to a greater extent in amyloid beta treated AD cybrids [67]. AD mitochondrial function predisposes cells to increased amyloid beta production and cell death.

## 5. Mitochondrial Interaction with AD Pathology

Further studies demonstrate mitochondrial function influences AD pathology. Treating fibroblasts from control subjects with a mitochondrial membrane potential uncoupler (CCCP) triggers tau phosphorylation at sites altered in AD [68]. Complex I inhibitors also initiate AD-like tau alterations. Chronic rotenone treatment in rat brain triggers tau hyperphosphorylation and aggregation [69]. Studies often utilize triple transgenic mice to model AD. Triple transgenic mice express mutated forms of APP, tau and presenilin 1, causing them to develop amyloid plaques and tau tangles. Studies in female triple transgenic mice observe mitochondrial dysfunction prior to amyloid plaque formation. Female triple transgenic mice eventually experience increased mitochondrial amyloid beta levels which may exacerbate mitochondrial dysfunction. However, female triple transgenic mice experience decreased COX activity and increased glycolytic rates prior to amyloidosis [70]. Overexpressing a form of mutant APP in mice also causes mitochondrial gene upregulation in the hippocampus long before amyloid plaque deposition. Most of these upregulated genes contribute to oxidative phosphorylation (OXPHOS) [71]. 

Studies question whether mitochondrial dysfunction triggers Alzheimer’s pathology. Fukui et al. deleted the *COX10* gene, which encodes a necessary COX assembly factor, in triple transgenic mice. *COX10* deletion inhibits COX assembly, causing loss of function. *COX10* deficient triple transgenic mice produce fewer amyloid plaques and amyloid beta than triple transgenic mice with functional COX [72]. This finding suggests loss of COX function reduces amyloid plaque production. However, it should be noted that loss of COX function via *COX10* deletion likely stimulates different responses than those elicited by defective functioning of intact COX. Additional studies are needed to more fully examine mitochondrial dysfunction’s effects on AD pathology.

A reciprocal relationship exists between AD pathology and mitochondrial function. Amyloid beta treatment in cell culture causes mitochondrial dysfunction, including decreases in membrane potential, electron transport chain activity and oxygen consumption [73]. Amyloid beta inhibits COX activity in isolated mitochondria [74]. In AD brains APP accumulates in mitochondrial translocases, potentially inhibiting their function [53]. Further work describes AD mitochondrial amyloid beta accumulation and interaction with an alcohol dehydrogenase within the mitochondrial matrix [75,76]. Tau also interacts with mitochondria and their biology. Tau overexpression in cell culture changes mitochondrial localization, likely by disrupting mitochondrial transport along microtubules. Post mortem AD brain studies observe decreased synaptic mitochondria suggesting AD disturbs neuronal mitochondrial transport [77]. Pathological tau may contribute to microtubule disruption and subsequent mitochondrial localization changes in AD. Hyperphosphorylated tau associates with voltage dependent anion channel 1 (VDAC1) on the outer mitochondrial membrane. AD increases hyperphosphorylated tau bound to VDAC1, another potential contributor to mitochondrial dysfunction [78].

Tau truncation also occurs in AD, potentially contributing to mitochondrial dysfunction. AD NFTs contain truncated tau and these truncated tau species may be toxic [79,80]. Overexpressing a specific N-terminal tau fragment (NH2-26-44) causes primary neurons to die. N-terminal tau fragment treatment inhibits adenine nucleotide transporter (ANT) function, causing mitochondrial dysfunction [81]. Further studies need to determine whether this N-terminal tau fragment increases during AD progression. Overexpressing another tau fragment (Asp-421 cleaved tau), known to increase during AD, causes mitochondrial fragmentation and increased oxidative stress in cell culture [82]. Tau fragment generation likely occurs through caspase cleavage during apoptosis. Additional AD-associated protein fragments disrupt mitochondrial function.

Apolipoprotein E allele ε4 (apoE4) increases risk for AD. Relative to other apoE isoforms, apoE4 accumulates in endosomal compartments and stimulates cholesterol efflux less efficiently [83]. Furthermore, apoE4 appears susceptible to c-terminal protease cleavage. C-terminal apoE fragments occur in AD brain and truncated apoE colocalizes with NFTs. Overexpressing apoE4 fragments (apoE4 Δ272–299) in cell culture stimulates NFT formation [84]. ApoE associates with mitochondrial proteins, with apoE4 fragments binding mitochondrial proteins more strongly than apoE2 and apoE3. Overexpressing apoE4 fragments decreases mitochondrial complex III and COX activity [85], suggesting apoE4 increases AD risk partly through mitochondrial effects.

## 6. Mitochondrial Contributions to Proteostasis

**Figure 1:**
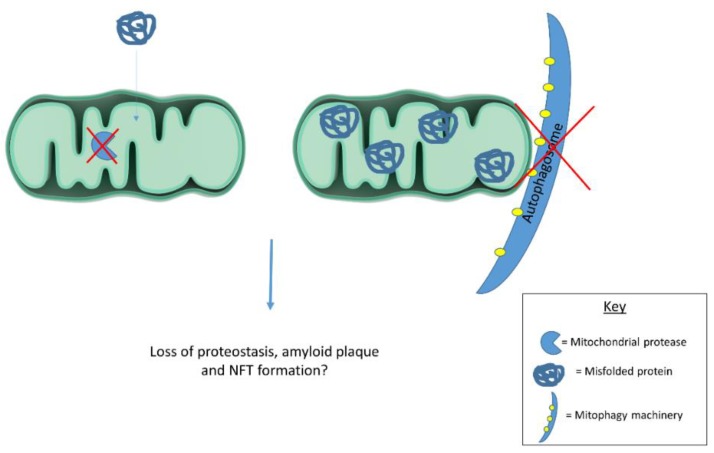
Mitochondrial proteases and mitophagy contribute to cellular proteostasis. Dysfunctional proteases and/or mitophagy could contribute to protein misfolding in disease states. Future work should examine mitochondrial contributions to cellular proteostasis in human cells as this process has largely been described in yeast.

Emerging evidence suggests mitochondria contribute to cellular proteostasis (Figure 1). In yeast, mitochondria degrade misfolded cytosolic proteins through resident proteases. Ruan et al. [86] show aggregated protein degradation in yeast relies on mitochondrial import machinery and proteases. When the authors blocked mitochondrial protein import and deleted mitochondrial proteases, protein aggregates became more stable. Defective cytosolic chaperones caused misfolded proteins to accumulate in mitochondria. Together, these observations highlight mitochondrial contributions to yeast proteostasis. The authors refer to mitochondrial protein degradation as “Mitochondria as Guardians in the Cytosol” (MAGIC) [86]. Whether MAGIC contributes substantially to proteostasis in human cells remains unclear. If MAGIC occurs in human cells, defective mitochondrial proteastasis could contribute to AD plaque and tangle formation. Another study shows mitochondrial degradation via mitophagy reduces amyloid burden in mAPP transgenic mice. mAPP mice lacking PTEN-induced putative kinase (PINK1) accumulate amyloid pathology earlier than mAPP mice expressing PINK1. PINK1 accumulation in mitochondrial membranes stimulates mitophagy. PINK1 knockout, therefore, seems to increase amyloid pathology in mAPP mice by disrupting mitophagy. Alternatively, PINK1 overexpression in mAPP mice enhances mitophagy and reduces amyloid beta plaques [87]. Mitophagy induction likely reduces mAPP mouse plaque burden by degrading amyloid beta filled mitochondria. In line with these findings, another report highlights mitophagy’s role in clearing protein aggregates. Findings suggest mitochondrial fission facilitates selective mitophagy of regions containing protein aggregates [88]. These studies suggest mitochondria act as disposal sites for aggregated proteins. Pathological protein aggregates may signal defective mitochondrial proteostasis or mitophagy.

Mitochondria possess intrinsic mechanisms for responding to unfolded proteins. Mitochondrial protein misfolding triggers a compensatory mechanism termed the mitochondrial unfolded response (mtUPR). Mutant ornithine transcarbamylase (OTC) overexpression leads misfolded OTC to accumulate within mitochondria, stimulating the mtUPR [89]. The mtUPR induces mitochondrial proteases and chaperones to restore proteostasis. Studies in *Caenorhabditis elegans* (*C. elegans*) provide most of the evidence for a mtUPR. mtDNA depletion by ethidium bromide, doxycycline treatment and mitochondrial ribosomal protein knockdown all trigger the mtUPR in *C. elegans*. Also, disrupting mitochondrial protein complexes and knocking down mitochondrial proteases and chaperones activates the mtUPR [90]. In *C. elegans*, Activating Transcription Factor associated with Stress-1 (ATFS-1) mediates mtUPR activation. ATFS-1 controls the mtUPR based on its subcellular localization. Functional mitochondria import and degrade ATFS-1. When mitochondrial dysfunction occurs, ATFS-1 accumulates in the nucleus due to a nuclear targeting sequence. Nuclear ATFS-1 activates mitochondrial protease and chaperone transcription. The mtUPR and ATFS-1 gained notoriety following discoveries of lifespan extension in *C. elegans* upon electron transport chain (ETC) gene knockdown [91]. Groups posited mtUPR activation mediates the lifespan extension gained from ETC gene knockdown. However, *C. elegans* lifespan studies suggest mtUPR activation and ATFS-1 activity do not facilitate the observed lifespan extension following mitochondrial insult [92].

In mammalian cells, activating transcription factor 5 (ATF5) may regulate an mtUPR similar to how ATFS-1 functions in *C. elegans*. However, distinct mitochondrial stress response pathways appear to predominate in mammalian cells. Studies examining diverse mitochondrial stressors suggest ATF5 activation occurs under specific circumstances. Paraquat treatment and mutant OTC overexpression stimulate mitochondrial chaperone and protease transcription in an ATF5-dependent manner. While ATF5 appears responsive to paraquat and mutant OTC, studies reveal activating transcription factor 4 (ATF4) responds to numerous mitochondrial stressors. Quiros et al. [93] introduced mammalian cells to distinct mitochondrial stressors, including membrane depolarization, translation inhibition, OXPHOS inhibition and protein import suppression. Mitochondrial stressors failed to induce either mtUPR or ATF5 activation, instead stimulating ATF4 dependent stress response pathways. Numerous studies implicate ATF4 in the mitochondrial stress response. ATF4 orchestrates diverse metabolic changes to help cells cope with mitochondrial dysfunction. Abrogating ATF4 decreases cellular proliferation, especially following mitochondrial stress [93]. In line with these findings, another study finds mitochondrial OXPHOS inhibitors stimulate stress response genes via ATF4 induction [94]. ATF4 clearly responds to diverse mitochondrial stressors, leading researchers to examine how mitochondrial dysfunction activates ATF4. While this review will focus on ATF4 related stress responses, mitochondrial dysfunction stimulates diverse compensatory mechanisms.

## 7. Mitochondrial Dysfunction Triggers Numerous Retrograde Responses, Including the Integrated Stress Response (mtISR)

Mitochondrial dysfunction triggers numerous changes in nuclear gene expression [95,96]. Referred to as retrograde responses, mitochondrial stress responses preserve cell viability by modulating metabolic pathways and mitochondrial function. Studies in *Saccharomyces cerevisiae* have elucidated mitochondrial-nuclear communication pathways in great detail [97]. While fewer studies on mammalian retrograde responses exist, certain pathways consistently respond to mitochondrial stressors. Multiple mitochondrial stressors perturb cytosolic calcium (Ca^2+^) and ROS levels, activating nuclear factor kappa B (NFκB). Although NFκB activation is canonically associated with immune system function, diverse cellular stressors, including mitochondrial dysfunction, activate NFκB [97]. Mitochondrial dysfunction activates NFκB in a manner distinct from cytokine mediated NFκB activation. Furthermore, NFκB may regulate c-Myc transcription, a transcription factor consistently upregulated by mitochondrial dysfunction [98,99]. c-Myc forms a Myc-Max heterodimer homologous to yeast retrograde response mediators [97]. NFκB and Myc activity increase in aged tissues and decrease during cell senescence [100]. Numerous cell signaling pathways participate in tightly orchestrated, context dependent retrograde responses. In yeast, retrograde responses facilitate replicative lifespan extension. Some groups speculate retrograde responses act similarly in mammalian cells to compensate for age-related mitochondrial deficits [101,102].

Many studies show that mitochondrial stress activates ATF4 signaling, suggesting ATF4 plays a role in retrograde signaling. ATF4 activation occurs through a pathway known as the integrated stress response (ISR). The ISR begins with eukaryotic initiation factor 2 alpha (eIF2α) phosphorylation [103]. Four kinases, heme-regulated inhibitor (HRI), protein kinase R (PKR), PKR-like endoplasmic reticulum kinase, (PERK) and general control non-depressible 2 (GCN2), phosphorylate eIF2α. Heme depletion, viral infection, endoplasmic reticulum stress, and amino acid starvation activate each kinase, respectively [104]. Studies implicate eIF2α phosphorylation in long term potentiation and long term memory through downstream effects on cyclic AMP responsive element binding protein (CREB), providing a potential link between the ISR and cognitive decline [105]. eIF2α phosphorylation triggers diverse cellular effects. 

One of eIF2α phosphorylation’s most important effects is to pause general protein translation, assisting in cellular stress recovery. However, eIF2α phosphorylation paradoxically increases protein translation from mRNAs possessing alternative open reading frames (ORFs). Numerous stress responsive factors contain alternative ORFs. Therefore, eIF2α phosphorylation reduces cell protein loads while preferentially increasing stress response factors [106]. ATF4 translation increases following eIF2α phosphorylation due to ATF4’s alternative ORFs [107]. Increased ATF4 translation stimulates downstream target transcription, including C/EBP homologous protein (CHOP) [108]. CHOP induction favors cell cycle arrest and, upon chronic activation, apoptosis [109,110]. In summary, the ISR responds to numerous stressors by reducing general protein translation while upregulating stress responsive factors. Major ISR mediators include eIF2α, ATF4, and CHOP. 

Several reports show mitochondrial dysfunction stimulates the ISR in mammalian cells. Rotenone treated oligodendroglia increase eIF2α phosphorylation as well as ATF4 and CHOP protein [111]. Earlier studies show CHOP mediates a mitochondrial specific stress response [112]. mtDNA depletion and doxycycline treatment in cell culture activate CHOP expression in an ATF4-dependent manner without concomitant mtUPR activation [113]. Multiple studies indicate mitochondrial protease inhibition specifically induces the ISR. Knocking out a mitochondrial serine protease, HtrA2, triggers the ISR in mouse brain [114]. Similarly, LON protease (LONP1) deficient cell lines exhibit mitochondrial protein aggregation along with ISR activation. LONP1 functions as an important mitochondrial matrix protease. Mitochondrial protein aggregates stemming from LONP1 depletion only modestly induce the mtUPR. The authors conclude LONP1 depletion prominently activates the ISR, while slightly increasing some mitochondrial proteases and chaperones [115].

Further studies show mitochondrial stress induces the ISR in muscle and brain tissue. Deletor mice possessing a dominant Twinkle (helicase involved in mtDNA replication) mutation model mitochondrial myopathy. Deletor mice rapidly accumulate mtDNA mutations leading to OXPHOS deficiency. OXPHOS deficiency in deletor mice triggers ISR components resulting in altered one carbon metabolism, serine synthesis, and glutathione production pathways (transulfuration) [116]. Quiros et al. note similar metabolic changes following mitochondrial dysfunction and ISR activation [93]. Furthermore, mammalian target of rapamycin complex 1 (mTORC1) inhibition in deletor mice rescues metabolic alterations by reducing ISR activity. In this model, mitochondrial dysfunction activates mTORC1 which subsequently activates the ISR [116].

Inducible Drp1 knockout in mouse neurons also stimulates the ISR. Drp1 knockout disrupts mitochondrial fission causing mitochondrial dysfunction and ISR activation. Drp1 knockout neurons increase fibroblast growth factor 21 (Fgf21) plasma protein and mRNA levels. A cytokine associated with mitochondrial myopathies, Fgf21 release increases upon mitochondrial dysfunction. Neuronal Drp1 knockout mouse studies show that brain mitochondrial dysfunction triggers Fgf21 release in an ISR dependent manner [117]. Some consider Fgf21 a mitokine, transmitting mitochondrial stress signals between organs [118]. ISR stimulation of Fgf21 expression further demonstrates a link between mitochondrial dysfunction and the ISR. However, the ISR responds to numerous cellular stressors. A mitochondrial stress-induced ISR refers to a unique ISR subgroup, a mitochondrial ISR (mtISR). Ample evidence of the mtISR exists, however, future research should examine signaling cascades stimulating the mtISR. 

As referenced earlier, numerous signals could activate ISRs. Heme depletion, viral infection, endoplasmic reticulum stress, and amino acid starvation all stimulate eIF2α kinases [104]. Determining specific signals responsible for the mtISR may prove difficult. Few studies associate mtISRs with specific eIF2α kinases. One study finds doxycycline treatment increases eIF2α phosphorylation through GCN2, the amino acid starvation sensitive kinase [113]. However, Quiros et al. knocked down all four eIF2α kinases following mitochondrial depolarization and saw no reductions in eIF2α phosphorylation. The authors concluded multiple kinases increase eIF2α phosphorylation during the mtISR [93]. Determining whether a mtISR occurs in sporadic diseases such as AD remains difficult since we do not know specific mtISR signatures. Numerous stressors occur in AD brain which may feed into the ISR. For example, endoplasmic reticulum (ER) stress occurs in AD and is known to strongly induce the ISR. Changes in ER calcium levels and protein glycosylation as well as misfolded protein accumulation trigger ER stress leading to eIF2α phosphorylation and increased ATF4 and CHOP [119,120]. ER stress activates another unique ISR subgroup, an ER stress-induced ISR (erISR). 

**Figure 2:**
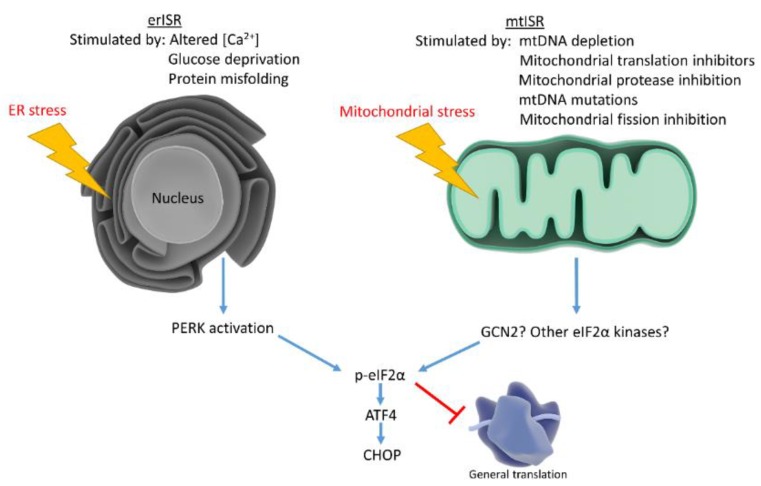
Mitochondrial stress and ER stress stimulate the ISR, which reduces general protein translation while upregulating stress responsive factors. ER stress activates the ISR through PERK, while mitochondrial stress may activate the ISR through multiple eIF2α kinases.

Mitochondria associate with the ER and assist in calcium maintenance, leading investigators to speculate whether mitochondrial dysfunction triggers ISR by causing ER stress. To determine whether mitochondrial dysfunction stimulates ER stress, studies examined classical ER stress markers not involved in the ISR. Studies found that mitochondrial dysfunction triggers the mtISR independently of general ER stress [93]. Although the mtISR does not appear to involve ER stress, these phenomena are not mutually exclusive (Figure 2). AD neurons exhibit increased ER stress markers concomitant with eIF2α phosphorylation. IHC studies show the ER stress markers, p-PERK and p-IRE1, increase in AD hippocampal neurons along with p-eIF2α. p-eIF2α and p-PERK antibodies stain similar granular structures in AD pyramidal hippocampal neurons, suggesting concomitant ER stress and ISR. While correlative, these findings suggest erISR occurs in AD [121]. Further work should attempt to determine whether a mtISR occurs in AD. To our knowledge, no articles discuss potential mitochondrial contributions to the observed AD ISR activation.

## 8. AD Activates the ISR: Could Mitochondrial Dysfunction Contribute?

Declining mitochondrial function during aging could theoretically stimulate retrograde responses [122]. AD brains display mitochondrial dysfunction beyond those observed with normal aging. Groups hypothesize that compensatory responses fail in AD once mitochondrial dysfunction passes a threshold, thus favoring disease progression [47]. In this hypothesized paradigm, beneficial compensatory responses decline during disease progression while potentially maladaptive stress responses predominate. Under certain conditions, ISR activation may represent a maladaptive response [123]. Maladaptive retrograde responses seem counterintuitive, however, inhibiting retrograde responses proves beneficial in multiple scenarios. For example, chronic ISR activation appears to favor cell death and inhibiting ISR activity proves beneficial in traumatic brain injury models [124]. Similarly, NFκB activation can favor apoptosis in cases of severe stress [125]. Researchers speculate that many retrograde responses may prove beneficial in the short term but become detrimental upon chronic activation [126].

Few studies focus on retrograde responses in the AD brain. In fact, many mammalian retrograde response studies use cancer cells which may respond differently to mitochondrial stress than postmitotic neurons. However, several lines of evidence suggest retrograde responses occur in neurons. *Drosophila melanogaster* models of neuronal mitochondrial dysfunction identify hypoxia inducible factor 1α, forkhead box O (FOXO) and ATF4 as key retrograde responders [127]. Human primary mitochondrial diseases often present with neurological deficits and cell lines carrying associated mtDNA mutations display retrograde responses [128]. Differentiated dopaminergic neurons treated with a complex I inhibitor upregulated ATF4 signaling pathways according to transcriptional profiling [129]. The diversity in retrograde responses makes it difficult to describe a canonical retrograde response in disease states, however, AD brains display changes in numerous factors implicated in mammalian retrograde responses.

Postmortem AD brains generally display NFκB activation and cyclic AMP response element binding protein (CREB) alterations, both of which can be affected by mitochondrial dysfunction and subsequent changes in Ca^2+^ concentration [130]. AD brains also possess decreased HIF1α and cortical SIRT1 levels [131,132]. The AD parietal lobe also displays activated Akt/mTOR as well as increases in downstream targets [133]. All of these factors can participate in retrograde responses, however, they all respond to diverse stimuli. AD mitochondrial dysfunction could activate these response or, alternatively, deficits in these responses could make cells more vulnerable to mitochondrial dysfunction. Currently, there is not enough evidence to support strong conclusions regarding the nature and function of retrograde responses in AD.

Another hypothetical consequence of AD mitochondrial dysfunction is ISR activation. Post-mortem AD brains display ISR activation. Given the well documented mitochondrial dysfunction in AD and evidence suggesting mitochondrial dysfunction stimulates the mtISR, it seems possible a mtISR occurs in AD brain. Numerous studies describe increased eIF2α phosphorylation in AD brains, particularly in hippocampal neurons [134,135] ATF4 protein levels increase in AD frontal cortex and increased ATF4 correlates fairly well with increased p-eIF2α [135]. IHC studies reveal increases in ATF4 positive cells in AD entorhinal cortex and subiculum, but decreases in the hippocampus [136]. CHOP protein also increases in AD cortex [137]. AD brains display defects in ribosome function and protein translation, although the ISR’s role in these deficits remains unclear [138]. Whether the ISR activation in AD stems largely from ER stress or mitochondrial dysfunction remains unknown.

Several groups propose amyloid beta and tau alterations trigger the ISR. In embryonic rat hippocampal cultures, amyloid beta oligomer treatment induces axonal ATF4 and CHOP synthesis. ATF4 siRNA desensitizes rat hippocampal cultures to amyloid beta’s negative effects, suggesting ATF4 potentiates amyloid beta toxicity [136]. However, aged Tg2576 mice, which accumulate amyloid plaques, do not display CHOP induction [137]. Colocalization experiments with ER stress markers and tau antibodies reveal a correlation between p-PERK staining and pretangle neurons containing hyperphosphorylated tau. Interestingly, neurons decorated with NFTs rarely display p-PERK staining, suggesting ER stress markers appear early in disease progression [139]. ER stress and mitochondrial dysfunction independently activate the ISR. Both ER stress and mitochondrial dysfunction occur in AD. Therefore, it seems reasonable that the mtISR may occur in AD alongside the erISR. While evidence suggests the possibility of a mtISR in AD, more work is needed to support this hypothetical relationship. If AD mitochondrial dysfunction triggers disease relevant retrograde responses, then therapeutic approaches should consider whether the compensatory mechanisms represent beneficial or maladaptive responses.

## 9. Conclusions

Mitochondrial dysfunction occurs in AD. Mitochondrial and metabolic abnormalities present early in disease progression. Systemic AD metabolic changes may prove useful diagnostically, and mitochondrial dysfunction seems to be a reasonable therapeutic target. Mitochondria-specific stress responses help cells cope with mitochondrial dysfunction. Certain mitochondrial stress response components are activated in AD, although to what extent these stress responses contribute to retarding or promoting AD progression remains unclear.
